# NURSING HOME STAFF’S PERCEPTIONS AND EXPERIENCES COLLABORATING WITH HOSPICE

**DOI:** 10.1093/geroni/igac059.2491

**Published:** 2022-12-20

**Authors:** Susanny Beltran, Norma Conner, Adam Reres, Morris Beato

**Affiliations:** University of Central Florida, Orlando, Florida, United States; University of Central Florida, Orlando, Florida, United States; University of Central Florida, Orlando, Florida, United States; University of Central Florida, Orlando, Florida, United States

## Abstract

Hospice has been associated with improved outcomes for terminally-ill patients and families, including in pain management, care satisfaction, and rates of hospitalizations. In 2016, 1/3 of Medicare hospice beneficiaries died in nursing homes (NH). The responsibilities for meeting the needs of the NH resident receiving hospice are shared by the NH staff and hospice team, making good communication and coordination of services between providers critical to the successful delivery of services. This exploratory study surveyed NH direct care and administrative staff about their perceptions of hospice, and barriers to collaboration. A total of 66 NH staff completed the online survey. The sample was 62.1% direct care staff (e.g., social work, nursing) and 37.8% administrators, predominately female (75%), non-Hispanic-white (43.9%), and employed full-time (87.8%). Over half of the NHs were non-profit organizations (56.1%). Respondents had on average 33.8 hours of hospice education and held positive hospice attitudes, with 76% strongly agreeing that hospice should be an option regardless of setting, and 87.8% believing they could collaborate with hospice toward the goal of a “good death.” Although participants cited barriers to hospice related to COVID-19, this was not associated with overall hospice attitudes. Respondents cited resistance from family as the primary barrier to hospice, indicating lack of knowledge and a need for family education. They also cited concerns surrounding duplication of roles. Family members in previous studies have identified care allocation as a challenge, based on expectations that hospice would bring additional contact/services instead of replacing NH services. Implications will be discussed.

